# Metformin Inhibits Angiotensin II-Induced Differentiation of Cardiac Fibroblasts into Myofibroblasts

**DOI:** 10.1371/journal.pone.0072120

**Published:** 2013-09-02

**Authors:** Jian Bai, Na Zhang, Ying Hua, Bingjian Wang, Lin Ling, Albert Ferro, Biao Xu

**Affiliations:** 1 Department of Cardiology, Drum Tower Hospital, Nanjing University Medical School, Nanjing, China; 2 Department of Clinical Pharmacology, Cardiovascular Division, King’s College London, London, United Kingdom; University of Illinois at Chicago, United States of America

## Abstract

Differentiation of cardiac fibroblasts into myofibroblasts is a critical event in the progression of cardiac fibrosis that leads to pathological cardiac remodeling. Metformin, an antidiabetic agent, exhibits a number of cardioprotective properties. However, much less is known regarding the effect of metformin on cardiac fibroblast differentiation. Thus, in the present study, we examined the effect of metformin on angiotensin (Ang) II-induced differentiation of cardiac fibroblasts into myofibroblasts and its underlying mechanism. Adult rat cardiac fibroblasts were stimulated with Ang II (100 nM) in the presence or absence of metformin (10–200 µM). Ang II stimulation induced the differentiation of cardiac fibroblasts into myofibroblasts, as indicated by increased expression of α-smooth muscle actin (α-SMA) and collagen types I and III, and this effect of Ang II was inhibited by pretreatment of cardiac fibroblasts with metformin. Metformin also decreased Ang II-induced reactive oxygen species (ROS) generation in cardiac fibroblasts via inhibiting the activation of the PKC-NADPH oxidase pathway. Further experiments using PKC inhibitor calphostin C and NADPH oxidase inhibitor apocynin confirmed that inhibition of the PKC-NADPH oxidase pathway markedly attenuated Ang II-induced ROS generation and myofibroblast differentiation. These data indicate that metformin inhibits Ang II-induced myofibroblast differentiation by suppressing ROS generation via the inhibition of the PKC-NADPH oxidase pathway in adult rat cardiac fibroblasts. Our results provide new mechanistic insights regarding the cardioprotective effects of metformin and provide an efficient therapeutic strategy to attenuate cardiac fibrosis.

## Introduction

Cardiac fibrosis is a critical aspect of cardiac remodeling following myocardial infarction, hypertension, and other cardiovascular diseases [1], [2]. Differentiation of cardiac fibroblasts into myofibroblasts, characterized by expression of α-smooth muscle actin (α-SMA) and production of extracellular matrix (ECM) components such as collagen types I and III, is a critical event in cardiac fibrosis [3], [4]. Angiotensin II (Ang II), which has been reported to be aberrantly activated in various cardiovascular diseases such as myocardial infarction and hypertension [5], [6], plays a major role in cardiac fibrosis by promoting myofibroblast differentiation [7]. Therefore, attenuation of Ang II-induced myofibroblast differentiation could be an important means for improving cardiac fibrosis.

Reactive oxygen species (ROS) are known to play an important role as signaling and regulatory molecules in cell apoptosis, migration, and differentiation [8], [9]. Several recent studies have reported that ROS play an important role in fibrotic diseases, including renal, liver and pulmonary fibrosis [10], [11], [12]. Since ROS are generated by Ang II stimulation in several cells [13], [14], it is possible that Ang II-induced myofibroblast differentiation is mediated via the activation of ROS production.

Metformin is a widely used antidiabetic agent that improves insulin sensitivity in patients with type 2 diabetes. The United Kingdom Prospective Diabetes Study (UKPDS) has shown that metformin therapy significantly reduces the risk of cardiovascular end points and all cause mortality in overweight type 2 diabetes patients, compared with other antidiabetic therapies [15]. Sasaki et al recently reported that metformin can prevent the progression of heart failure in dogs along with the activation of AMPK [16]. These studies suggest that metformin has additional cardioprotective effects beyond its antihyperglycemic properties.

Inhibition of cardiac fibrosis may be one of the cardioprotective effects of metformin. Xiao and colleagues have reported that metformin inhibits pressure overload-induced cardiac fibrosis in vivo and reduces collagen synthesis in cardiac fibroblasts [17]. Furthermore, metformin has been found to attenuate cardiac fibrosis in nondiabetic rats with post-myocardial infarction heart failure [18]. These studies strongly suggest that metformin can attenuate cardiac fibrosis in a variety of cardiovascular diseases. However, little is known about the role of metformin in Ang II-induced differentiation of cardiac fibroblasts into myofibroblasts.

Thus, in the present study, we investigated the effect of metformin on Ang II-induced myofibroblast differentiation and our data indicate that metformin inhibits Ang II-induced myofibroblast differentiation by suppressing ROS generation via the inhibition of the PKC-NADPH oxidase pathway in adult rat cardiac fibroblasts.

## Materials and Methods

### Ethics Statement

Animal experiments conformed to the Guide for the Care and Use of Laboratory Animals published by the US National Institutes of Health (NIH Publication No. 85-23, revised 1996) and was approved by the Ethics Review Board for Animal Studies of Nanjing Drum Tower Hospital (DTH ERBA 66.01/023B/2011). Hearts were obtained from 8- to 10-week-old male Sprague-Dawley rats, which were sacrificed by cervical dislocation.

### Cell Culture

Adult rat cardiac fibroblasts were isolated as previously described [19]. Briefly, hearts were excised from 8- to 10-week-old male Sprague-Dawley rats. After rinsing in cold PBS, the ventricles were minced and digested in DMEM containing 0.1% collagenase type 2 (Worthington) at 37°C with continuous shaking for 30 minutes. The dissociated cells were collected and plated for 1h at 37°C. After pre-plating the unattached cells (including myocytes and endothelial cells) were removed and the cardiac fibroblasts were cultured in Dulbecco’s modified Eagle’s medium (DMEM) containing 10% foetal bovine serum, 100 U/ml penicillin and 100 µg/ml streptomycin. Using this method, we obtained >95% cardiac fibroblasts at passage 1 as detected by positive staining for vimentin. Cells in the passage 2 were used in this study. Cardiac fibroblasts were treated with Ang II (100 nM) for 12 or 24 h alone or pretreated with metformin in different concentrations (10, 50, and 200 µM) for 1 h before treatment with Ang II. In some experiments, cardiac fibroblasts were pretreated with 1 mM apocynin (Sigma) or 0.1 µM calphostin C (Sigma) for 1 h.

### Measurement of Intracellular ROS

Intracellular ROS generation was detected using the fluorescent probe 2′, 7′-dichlorofluorescin diacetate (DCFH-DA) as previously described [20]. Briefly, cardiac fibroblasts were plated in in six-well plates at a density of 1 × 105 cells/well. After different treatments, medium was removed, and the cells were washed with serum-free medium twice. A solution of 5 µM DCFH-DA probe in serum free media was then added for 30 min at 37°C. After the incubation, the ROS signal in cells was detected by flow cytometry (FACSCanto, Becton Dickinson) and confocal microscope (FluoView 1000, Olympus).

### NADPH Oxidase Activity Assay

Cardiac fibroblasts were washed with ice-cold PBS, collected by a cell scraper, and homogenized in lysis buffer. NADPH oxidase activity of the cell lysate was measured using cell NADPH oxidase colorimetric assay kit (Genmed Scientific Inc.) according to the manufacturer’s instructions. Briefly, NADPH oxidase activity was measured by monitoring the rate of consumption of NADPH (decrease in absorbance at 340 nm for 5 min) that was inhibited by the addition of DPI. Results were expressed as a percentage of the control value.

### Immunofluorescence Staining

Cardiac fibroblasts cultured on glass coverslips were fixed in 4% paraformaldehyde for 10 min, washed with PBS 3 times, and permeabilized with 0.1% Triton X-100 in PBS for 20 min. After rinsing with PBS 3 times, the slides were blocked with 1% bovine serum albumin at room temperature for 30 min. Next, the cells were incubated with a rabbit polyclonal anti-α-smooth muscle actin primary antibody (1∶100 dilution, Abcam) overnight in a humidified incubator. The slides were then rinsed and incubated with a goat anti-rabbit secondary antibody conjugated to Alexa Fluor 488 (1∶400 dilution, Invitrogen) for 1 h. Finally, the slides were counterstained with DAPI (Sigma) for 10 minutes, mounted in fluoromount-G (Southern Biotech), and analyzed with a Fluoview 1000 confocal microscope.

### Western Blotting

For protein analysis, the cells were lysed in RIPA buffer containing 10 mM Tris-HCl (pH 7.2), 150 mM NaCl, 0.1% SDS, 1% sodium deoxycholate, 5 mM EDTA, 1% TritonX-100, 50 mM NaF, 1 mM Na3VO4, and protease inhibitor cocktail (Roche). Protein concentrations were determined by BCA protein assay kit (Pierce). Equal protein amounts of the sample (30 µg) were separated on a 10% SDS-polyacrylamide gel and then transferred to PVDF membranes (Millipore). The membranes were blocked in 5% nonfat dry milk in TBS-Tween and then incubated with primary antibodies overnight at 4°C. Primary antibodies used were mouse anti-α-SMA (1∶1000, Abcam) and anti-β-actin (1∶1000, Abcam). After washing three times with TBS-Tween, the membranes were incubated with horseradish peroxidase-conjugated anti-mouse IgG for 2 h at room temperature. The immunoreactive bands were visualized by enhanced chemiluminescence detection system (Pierce), and band densities were quantified for each band following scanning using Quantity One Software (Bio-Rad).

For the study of PKC translocation, the membrane and cytosolic fractions were prepared by the Mem-PER Eukaryotic Membrane Protein Extraction Reagent Kit (Pierce) according to the manufacturer’s instructions. Primary antibodies used were rabbit anti-PKC α (1∶1000, Bioworld Technology), anti-PKC β (1∶500, Bioworld Technology), anti-PKC δ (1∶500, Bioworld Technology) and anti-PKC ε (1∶500, Bioworld Technology).

### Real-Time PCR

As previously described [21], total RNA was extracted by using the TRIzol method (Invitrogen) and dissolved in DEPC-treated water. Two micrograms of total RNA was reverse-transcribed into cDNA using the M-MLV reverse transcriptase (Invitrogen), according to the manufacturer's instructions. Real-time polymerase chain reaction was performed with a 7500 Real-Time PCR system (Applied Biosystems, CA, USA) using SYBR Premix Ex TaqTM (TaKaRa, Dalian, China). The reaction was performed at 95°C for 30 s, followed by 40 cycles of 95°C for 5 s and 60°C for 34 s. The dissociation stage was initiated at 95°C for 15 s, followed by 1 cycle of 60°C for 1 min and 95°C for 15 s. The relative gene expression for each sample was normalized to the expression of the housekeeping gene β-actin and analyzed by the 2^–ΔΔCt^ method. The primers for β-actin, collagen types Iand III are listed in Table 1.

**Table 1:** Primers used for RT-PCR.

Gene	Primer sequence (5′-3′)	Product size
collagen type I	Forward: TTCACCTACAGCACGCTTGT	196 bp
	Reverse: TTGGGATGGAGGGAGTTTAC	
collagen type III	Forward: GGTCACTTTCACTGGTTGACGA	201 bp
	Reverse: TTGAATATCAAACACGCAAGGC	
β-actin	Forward: AGACCTTCAACACCCCAG	254 bp
	Reverse: CACGATTTCCCTCTCAGC	

### Statistical Analysis

All data were presented as mean ± SEM. Comparisons between groups were performed by the non-parametric Kruskal-Wallis test followed by Mann-Whitney *U* tests. All data analysis was performed with the use of SPSS 13.0 statistical software. Statistical significance was defined as *P*<0.05.

## Results

### Metformin Inhibits Ang II-Induced Differentiation of Cardiac Fibroblasts into Myofibroblasts

**Figure 1:**
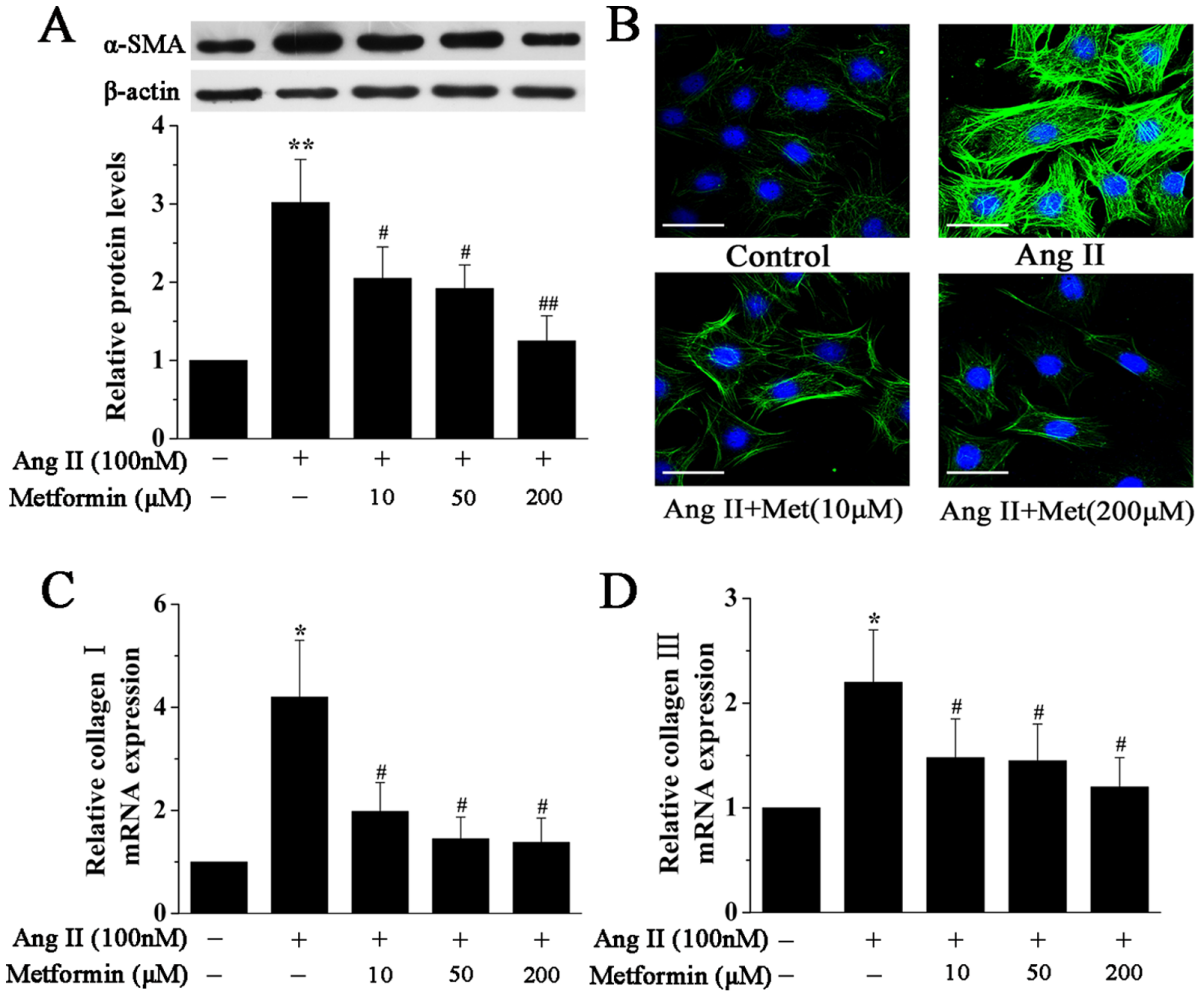
Metformin inhibits Ang II-induced cardiac myofibroblast differentiation. **A**, Representative Western blotting (upper panel) and quantitative analysis (lower panel) of α-SMA protein expression in cardiac fibroblasts treated with Ang II (100 nM) for 24 h in the presence or absence of metformin (10, 50, and 200 µM). α-SMA expression was normalized to β-actin. **B**, Representative images of immunofluorescence staining for α-SMA (green) in cardiac fibroblasts treated as above. Nuclei were stained with DAPI (blue). Scale bar, 50 µm. **C** and **D**, Real-time PCR quantification of Collagen I (A) and Collagen III (B) expression in cardiac fibroblasts treated with Ang II (100 nM) for 12 h in the presence or absence of metformin (10, 50, and 200 µM). The mRNA expression was normalized to corresponding β-actin mRNA. Data represent mean ± SEM of 4 separate experiments. *P<0.05 vs control group, *^#^P*<0.05 vs Ang II group, **P<0.01 vs control group, *^##^P*<0.01 vs Ang II group.

To evaluate myofibroblast differentiation, Western blotting and immunofluorescence staining were used to detect α-smooth muscle actin (α-SMA) expression. Cardiac fibroblasts were pretreated with metformin in different concentrations (10, 50, and 200 µM) for 1 h and then stimulated with Ang II (100 nM) for 24 h. As shown in Figure 1A, Ang II significantly enhanced α-SMA expression, and metformin pretreatment inhibited the expression of α-SMA as quantified by Western blotting. This effect was also observed by immunofluorescence analysis (Figure 1B). In addition, immunofluorescence staining also revealed an increased cell size of fibroblasts stimulated with Ang II compared with control, which was significantly reduced by metformin pretreatment.

### Metformin Inhibits Ang II-Induced Expression of Collagen I and Collagen III in Cardiac Fibroblasts

Compared with control, Ang II treatment increased the mRNA levels of collagen I and collagen III in cultured cardiac fibroblasts (Figure 1C and 1D), and the maximal mRNA levels were observed at 12 h after exposure to Ang II. Metformin also prevented the increase in Ang II-induced mRNA levels of collagen I and collagen III, as assessed by real-time PCR (Figure 1C and 1D).

### Metformin Inhibits Ang II-Induced ROS Generation and NADPH oxidase Activity in Cardiac Fibroblasts

**Figure 2:**
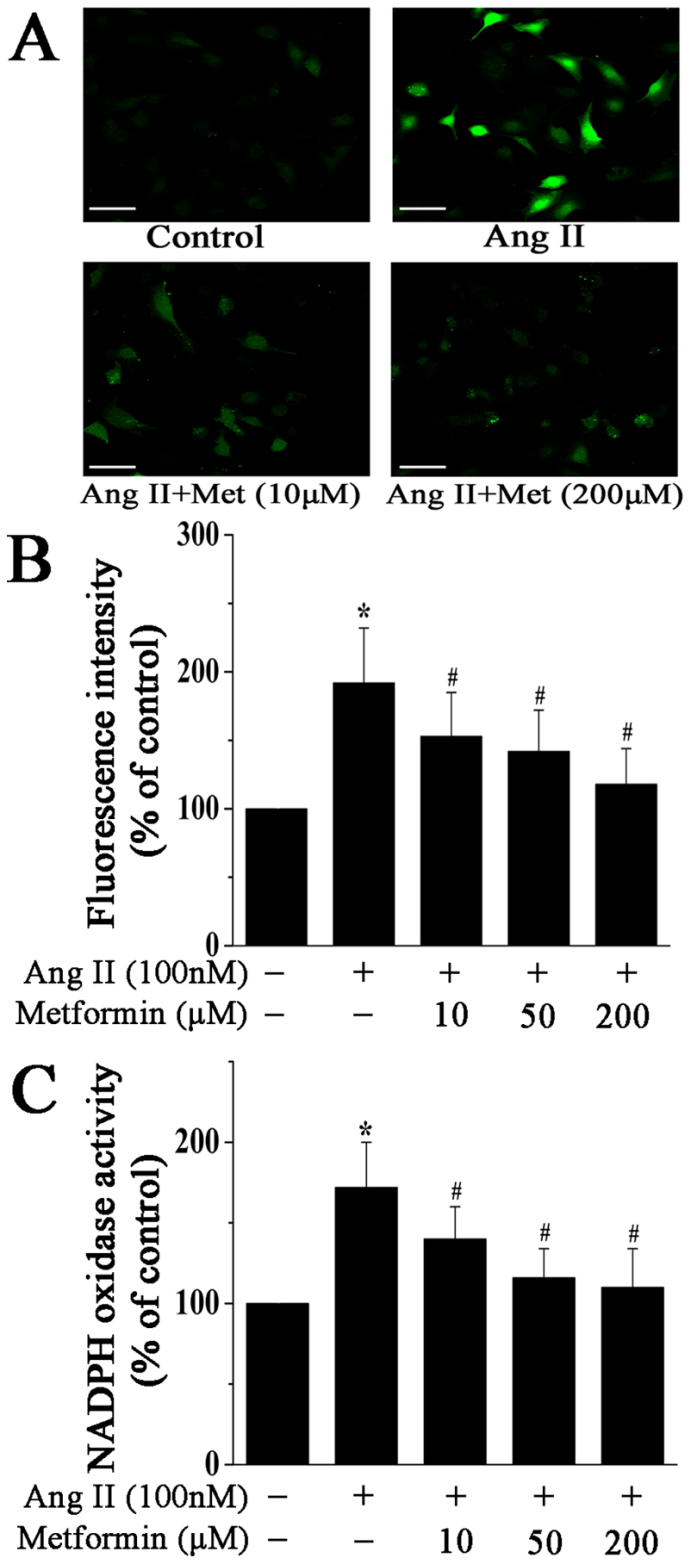
Metformin inhibits Ang II-induced ROS generation and NADPH oxidase activity in cardiac fibroblasts. **A**, Representative images of DCF fluorescence (green) in cardiac fibroblasts treated with Ang II (100 nM) for 12 h in the presence or absence of metformin (10 and 200 µM). Scale bar, 100 µm. **B**, Quantitative analysis of DCF fluorescence intensity by flow cytometry. **C**, Quantitative analysis of NADPH oxidase activity in cardiac fibroblasts treated as above. Data represent mean ± SEM of 5 separate experiments. *P<0.05 vs control group, *^#^P*<0.05 vs Ang II group.

Since ROS have been reported to mediate the differentiation of cardiac fibroblasts into myofibroblasts [22], we first investigated whether metformin could inhibit ROS generation in cardiac fibroblasts exposed to Ang II. Cardiac fibroblasts were pretreated with metformin in different concentrations for 1 h and then stimulated with Ang II (100 nM) for 12 h. After treatment, intracellular ROS generation was measured using a DCFH-DA method and analyzed by flow cytometry and confocal microscopy. As shown in Figure 2A and 2B, stimulation with Ang II markedly increased ROS generation in cardiac fibroblasts, which was attenuated by pretreatment with metformin.

We next examined the underlying mechanism of ROS generation by measuring the activity of NADPH oxidase, which is considered to be the main source of ROS in the cardiovascular system. Compared with control, stimulation with Ang II for 12 h significantly increased the activity of NADPH oxidase in fibroblasts, which was inhibited by metformin pretreatment (Figure 2C). These results indicate that metformin decreases Ang II-induced ROS generation in cardiac fibroblasts via inhibiting the activation of NADPH oxidase.

### Metformin Prevents Ang II-Induced Activation of PKC in Cardiac Fibroblasts

**Figure 3:**
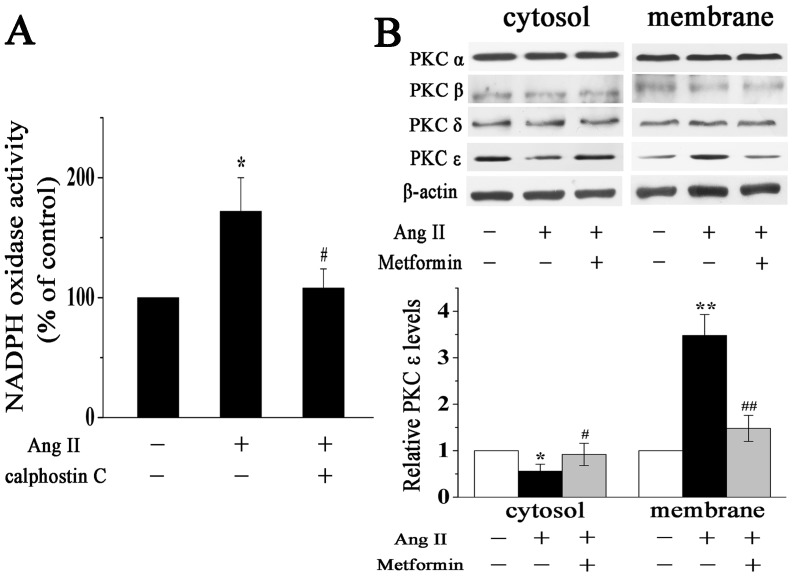
Metformin prevents Ang II-induced activation of PKC in cardiac fibroblasts. **A**, PKC mediates Ang II-induced activation of NADPH oxidase. Quantitative analysis of NADPH oxidase activity in cardiac fibroblasts treated with Ang II (100 nM) for 12 h in the presence or absence of PKC inhibitor calphostin C. **B**, Representative Western blotting (upper panel) and quantitative analysis (lower panel) of PKC isoforms expression in the cytosolic and membrane fractions of cardiac fibroblasts treated with Ang II (100 nM) for 12 h in the presence or absence of metformin (200 µM). PKC ε expression was normalized to β-actin. Data represent mean ± SEM of 5 separate experiments. *P<0.05 vs control group, *^#^P*<0.05 vs Ang II group, **P<0.01 vs control group, *^##^P*<0.01 vs Ang II group.

Since previous studies have clearly demonstrated that PKC mediates the activation of NADPH oxidase in various cell types [13], [14], we examined the role of PKC in Ang II-induced activation of NADPH oxidase in cardiac fibroblasts. Preincubation of cardiac fibroblasts with 0.1 µM calphostin C (a PKC inhibitor) abolished the Ang II-induced increase in NADPH oxidase activity (Figure 3A). Hence, Ang II-induced activation of NADPH oxidase in cardiac fibroblasts is mediated by PKC.

We next investigated the effect of metformin on Ang II-induced PKC activation in cardiac fibroblasts. PKC activation was assessed by determining the translocation of PKC from the cytosol to the membrane in cardiac fibroblasts as detected by Western blotting. As shown in Figure 3B, incubation with Ang II for 12 h induced a significant translocation of PKC ε from the cytosol to the membrane, which was restored by metformin (200 µM) pretreatment. However, translocation of PKC α, β and δ did not show a significant change, which is in line with the study by Stawowy et al [23]. Taken together, these results demonstrate that metformin decreases Ang II-induced activation of NADPH oxidase via inhibiting the activation of PKC in cardiac fibroblasts.

### The PKC-NADPH Oxidase Pathway Mediates Ang II-Induced Cardiac Myofibroblast Differentiation and Collagen Expression

**Figure 4:**
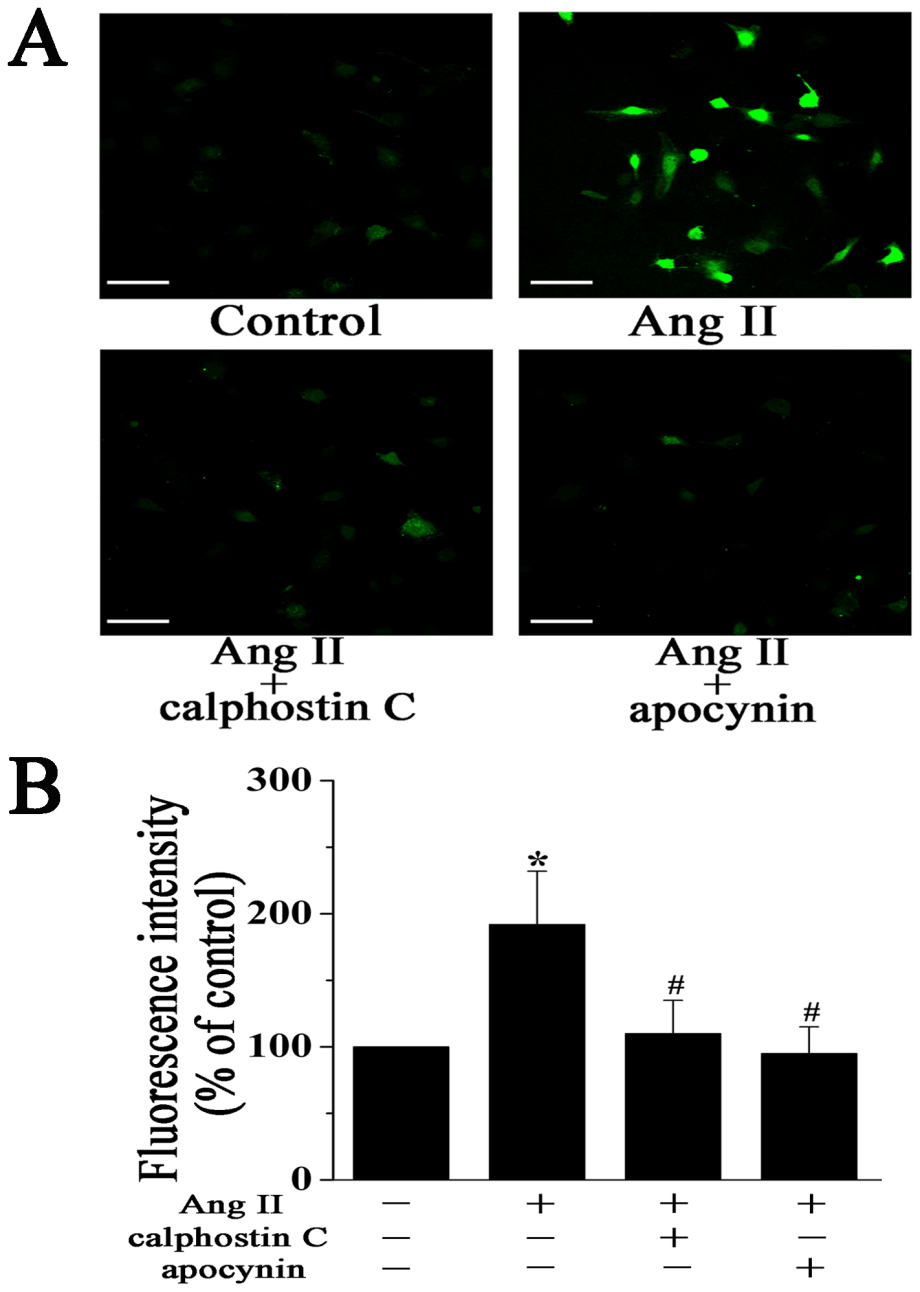
The PKC-NADPH oxidase pathway mediates Ang II-induced ROS generation in cardiac fibroblasts. **A**, Representative images of DCF fluorescence (green) in cardiac fibroblasts treated with Ang II (100 nM) for 12 h in the presence or absence of 0.1 µM calphostin C (PKC inhibitor) or 1 mM apocynin (NADPH oxidase inhibitor). Scale bar, 100 µm. **B**, Quantitative analysis of DCF fluorescence intensity by flow cytometry. Data represent mean ± SEM of 5 separate experiments. *P<0.05 vs control group, *^#^P*<0.05 vs Ang II group.

Based on these observations, we performed additional studies to determine whether the PKC-NADPH oxidase pathway is necessary for Ang II-induced cardiac myofibroblast differentiation and collagen expression. Cardiac fibroblasts were pretreated with PKC inhibitor calphostin C (0.1 µM) or NADPH oxidase inhibitor apocynin (1 mM) for 1 h and then incubated with Ang II. DCF analysis showed that treatment with calphostin C or apocynin markedly inhibited ROS generation induced by Ang II (Figure 4A and 4B).

**Figure 5:**
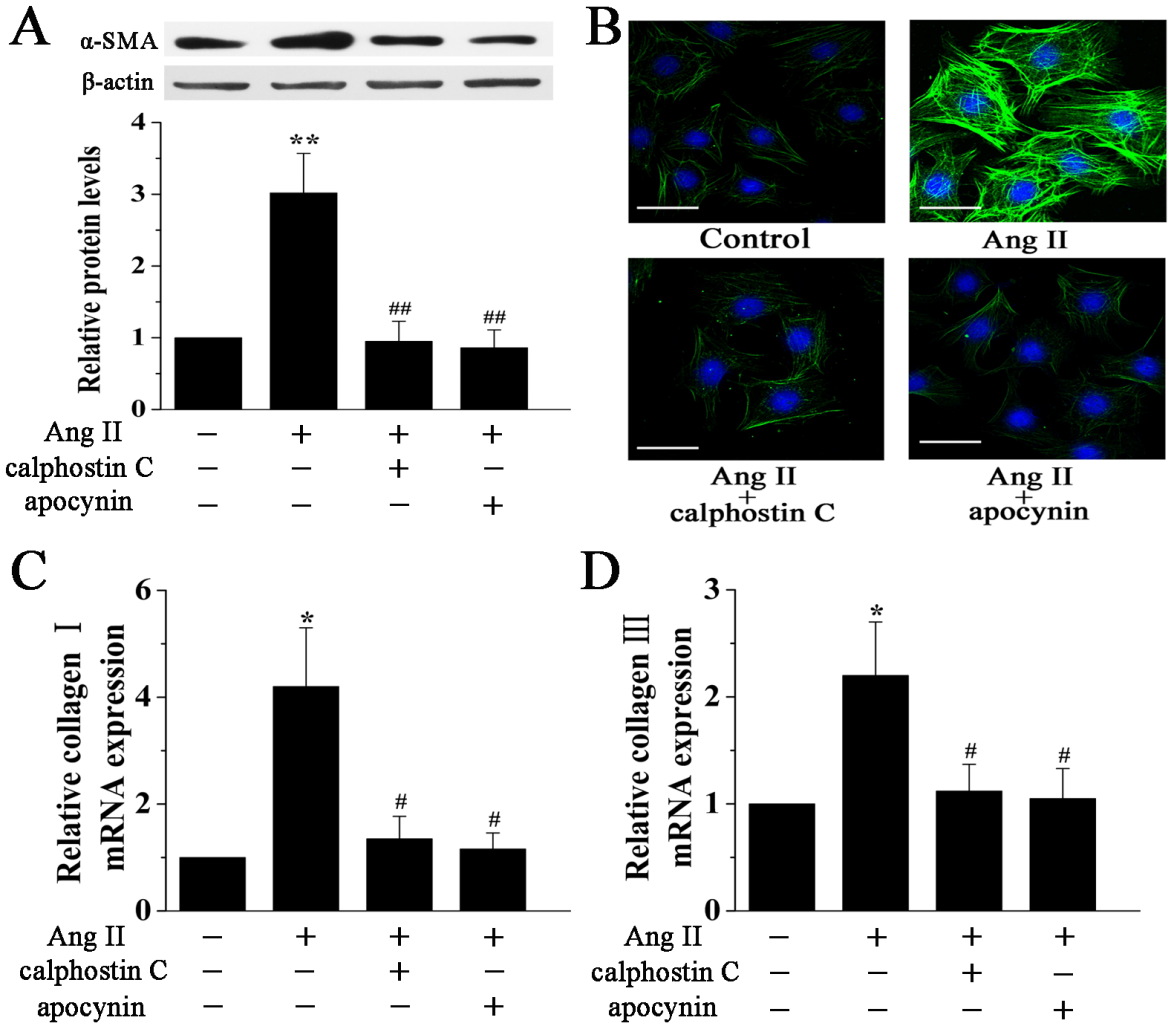
The PKC-NADPH oxidase pathway mediates Ang II-induced cardiac myofibroblast differentiation. **A**, Representative Western blotting (upper panel) and quantitative analysis (lower panel) of α-SMA protein expression in cardiac fibroblasts treated with Ang II (100 nM) for 24 h in the presence or absence of calphostin C (0.1 µM) or apocynin (1 mM). α-SMA expression was normalized to β-actin. **B**, Representative images of immunofluorescence staining for α-SMA (green) in cardiac fibroblasts treated as above. Nuclei were stained with DAPI (blue). Scale bar, 50 µm. **C** and **D**, Real-time PCR quantification of Collagen I (A) and Collagen III (B) expression in cardiac fibroblasts treated with Ang II (100 nM) for 12 h in the presence or absence of calphostin C (0.1 µM) or apocynin (1 mM). The mRNA expression was normalized to corresponding β-actin mRNA. Data represent mean ± SEM of 4 separate experiments. *P<0.05 vs control group, *^#^P*<0.05 vs Ang II group, **P<0.01 vs control group, *^##^P*<0.01 vs Ang II group.

Expression of α-SMA is associated with the differentiation of cardiac fibroblasts into myofibroblasts. Our data showed that treatment of cardiac fibroblasts with calphostin C or apocynin significantly reduced Ang II-induced increase in α-SMA protein expression (Figure 5A and 5B). Furthermore, the size of cardiac fibroblasts was also reduced by the treatment of calphostin C or apocynin, as assessed by immunofluorescence staining (Figure 5B). Another important effect of Ang II on cardiac fibroblasts is the stimulation of collagen synthesis. As shown in Figure 5C and 5D, treatment with calphostin C or apocynin significantly suppressed the mRNA levels of Collagen I and Collagen III induced by Ang II. Taken together, these results demonstrate that the PKC-NADPH oxidase pathway is critical for myofibroblast differentiation in response to Ang II.

## Discussion

In the present study, we demonstrate that metformin inhibits Ang II-induced myofibroblast differentiation in adult rat cardiac fibroblasts, and this may offer a new strategy to inhibit the process of cardiac fibrosis. Our results also show that the PKC-NADPH oxidase-derived ROS are essential for myofibroblast differentiation in adult rat cardiac fibroblasts in response to Ang II. Furthermore, metformin decreases Ang II-induced ROS generation in cardiac fibroblasts via inhibiting the activation of the PKC-NADPH oxidase pathway.

Differentiation of cardiac fibroblasts to myofibroblasts is characterized by expression of α-SMA and production of ECM components such as collagen types I and III [24], and activated myofibroblasts are considered the major source of ECM components that accumulate during cardiac fibrosis [25]. Ang II is produced by the enzymatic cascade involved in the renin-angiotensin system (RAS) and considered as a potent pro-fibrotic factor in the cardiovascular system via the activation of AT_1_ receptor [26]. Previous studies have reported that Ang II can stimulate cardiac myofibroblast differentiation and collagen synthesis *in vitro* and *in vivo* [27], [28], [29]. Since metformin has been shown to attenuate cardiac fibrosis in a dog model of heart failure [16], we have addressed whether metformin affects Ang II-induced myofibroblast differentiation *in vitro*. We observed that pretreatment of cardiac fibroblasts with metformin significantly inhibited Ang II-induced α-SMA protein expression as well as the expression of collagen types I and III. Our results suggest that metformin blunts cardiac fibrosis by inhibiting myofibroblast differentiation.

There is increasing evidence that oxidative stress plays an important role in pathological cardiac remodeling [30]. Cucoranu et al reported that ROS are necessary for α-SMA protein expression and myofibroblast differentiation in response to TGF-β1 [22]. In our study, we observed that Ang II significantly increased intracellular ROS generation. This observation is in line with these reported by other investigators [31], [32], [33]. It is well known that Ang II stimulates ROS generation in the cardiovascular system mainly via the activation of NADPH oxidase [34]. However, the signaling mechanisms by which Ang II activates NADPH oxidase in cardiac fibroblasts are not fully understood. Previous studies have shown that PKC plays a critical role in Ang II-induced activation of NADPH oxidase in cardiac myocytes and vascular smooth muscle cells [13], [14]. In the present study, we used different antagonistic strategies to elucidate the function of the PKC-NADPH oxidase pathway in regulating Ang II-induced myofibroblast differentiation and collagen synthesis. We found that PKC inhibitor calphostin C and NADPH oxidase inhibitor apocynin markedly inhibited the Ang II-induced increase in ROS generation in cardiac fibroblasts. We also noted that Ang II-induced activation of NADPH oxidase was prevented by PKC inhibition. Moreover, inhibition of PKC or NADPH oxidase could prevent Ang II induced α-SMA protein expression as well as the expression of collagen types I and III in cardiac fibroblasts. These data support the notion that Ang II mediated ROS generation, which is dependent on the PKC-NADPH oxidase pathway, is vital for the differentiation of cardiac fibroblasts into myofibroblasts. The mechanisms by which ROS mediate the differentiation of cardiac fibroblasts into myofibroblasts remain unclear. Accumulating evidence suggests that ROS can serve as signaling molecules to mediate the phosphorylation of ERK 1/2 and Smad [35], [36], which were associated with the differentiation of fibroblasts, derived from heart, lung and kidney, into myofibroblasts [37]. However, the precise underlying mechanism requires further investigation.

Metformin has previously been shown to reduce intracelluar ROS production in Ang II or high glucose-stimulated endothelial cells [38], [39]. However, the effect of metformin on Ang II-stimulated ROS generation in cardiac fibroblasts was unclear before our current study. In the present study, we found that metformin significantly reduced Ang II-induced ROS generation in cardiac fibroblasts. To further elucidate the mechanism, we examined the influence of metformin on the PKC-NADPH oxidase pathway. Our study showed that Ang II significantly induced the activation of PKC ε in cardiac fibroblasts, which is in accordance with previous study [23]. Pretreatment of cardiac fibroblasts with metformin effectively inhibited Ang II-induced activation of PKC ε and its downstream target NADPH oxidase. Our findings suggest that metformin reduces Ang II-induced ROS generation in cardiac fibroblasts by inhibiting the PKC-NADPH oxidase pathway. Recently, AMPK has been reported to suppress the activation of PKC in endothelial cells [40], [41]. We postulate that the inhibition of PKC activity by metformin may result from an activation of AMPK, but this remains to be defined.

In summary, our study demonstrates that metformin inhibits Ang II-induced myofibroblast differentiation by suppressing ROS generation via the inhibition of the PKC-NADPH oxidase pathway in adult rat cardiac fibroblasts, thus providing new mechanistic insights regarding the cardioprotective effects of metformin. Furthermore, the PKC-NADPH oxidase-ROS pathway plays an essential role in Ang II-induced myofibroblast differentiation and may offer potential targets for the treatment of cardiac fibrosis and its sequelae.
